# Effect of acupuncture on hot flush and menopause symptoms in breast cancer- A systematic review and meta-analysis

**DOI:** 10.1371/journal.pone.0180918

**Published:** 2017-08-22

**Authors:** Tsai-Ju Chien, Chung-Hua Hsu, Chia-Yu Liu, Ching-Ju Fang

**Affiliations:** 1 Institute of Traditional Medicine, National Yang-Ming University, Taipei, Taiwan; 2 Division of Hemato-Oncology, Department of Internal Medicine, Branch of Zhong-xing, Taipei City Hospital, Taipei, Taiwan; 3 Branch of Linsen, Chinese Medicine and Kunming, Taipei City Hospital, Taipei, Taiwan; 4 School of Chinese Medicine, College of Chinese Medicine, China Medical University, Taichung, Taiwan; 5 Medical Library, National Cheng Kung University, Tainan, Taiwan; Stanford University School of Medicine, UNITED STATES

## Abstract

**Background:**

Many breast cancer patients suffer from hot flush and medical menopause as side effects of treatment. Some patients undergo acupuncture, rather than hormone therapy, to relieve these symptoms, but the efficacy of acupuncture is uncertain. This meta-analysis evaluated the efficacy of acupuncture on hot flush and menopause symptoms in women with breast cancer.

**Methods:**

A literature search was performed, following the PRISMA Statement and without language restrictions, of 7 databases from inception through March 2017. All selected studies were randomized clinical trials (RCTs) that examined the effect of needle acupuncture on hot flush and menopause symptoms in patients with breast cancer. The methodological quality of these trials was assessed using Cochrane criteria, and meta-analysis software (RevMan 5.2) was used to analyze the data.

**Results:**

We examined 844 breast cancer patients (average age: 58 years-old) from 13 RCTs. The trials had medium-to-high quality, based on the modified Jadad scale. The meta-analysis showed that acupuncture had no significant effect on the frequency and the severity of hot flush (*p* = 0.34; *p* = 0.33), but significantly ameliorated menopause symptoms (*p* = 0.009). None of the studies reported severe adverse events.

**Conclusions:**

Acupuncture significantly alleviated menopause symptoms, but had no effect on hot flush. Breast cancer patients concerned about the adverse effects of hormone therapy should consider acupuncture. Further large-scale studies that also measure biomarkers or cytokines may help to elucidate the mechanism by which acupuncture alleviates menopause symptoms in patients with breast cancer.

## Introduction

Modern treatment modalities for breast cancer, including surgery, targeted therapy, and chemotherapy, have allowed patients to survive longer than previously. However, more than 60% of survivors develop climacteric syndrome, with symptoms such as hot flush[1, 2]. The prevalence of menopause-related sleep disturbances also range from 18.6–56.6%[3–5]. These symptoms can be clinically significant, negatively impact quality of life, and limit daily activities.

Hormone therapy (HT) is considered the most effective treatment for hot flush, but this treatment is often unsuitable for breast cancer survivors because many of them have already undergone HT, and exposure to estrogen increases the risk of breast cancer recurrence and cardiovascular disease[6–8]. Accordingly, many breast cancer patients with climacteric syndrome seek a complementary and alternative medicine (CAM) to relieve their symptoms, such as special diets, yoga, herbal therapies, acupuncture, and others[9, 10].

The mechanism by which acupuncture leads to physiological or clinical changes is still unclear, but it is widely accepted as a safe treatment[11]. Moreover, the understanding and practice of real-world acupuncture varies considerably. On the one hand, traditional Chinese medicine proposes that acupuncture alleviates pain and treats symptoms by regulating meridian energy (Qi); on the other hand, modern Western medicine has examined the mechanism of acupuncture based on changes in neurophysiologic and neuro-hormonal activities[12]. Some studies suggest that acupuncture increases endorphin activity, thereby modulating thermoregulation in the hypothalamus, and counteracting the disturbed thermoregulation of patients suffering from vasomotor syndromes[13]. Aside from the unknown mechanism and diverse theories of acupuncture[14], 5 to 71% of breast cancer patients choose to undergo acupuncture[15, 16] due to its safety, high accessibility, and the minimal risk of causing endometrial problems.

Recent studies have examined the effect of acupuncture on alleviation of hot flush and menopause syndromes in breast cancer patients. In recent years, some randomized controlled trials (RCTs) investigated the efficacy of acupuncture in reducing hot flush, sleeping disorders, quality of life, joint pain, and other symptoms of breast cancer patients[17, 18]. Different studies have used different control treatments (sham acupuncture, hormone therapy, applied relaxation, etc.) for comparison, to determine whether acupuncture outperforms other kinds therapy, or if the benefit is only due to the placebo effect. Although the data tends to show that acupuncture has a positive effect, the results are not totally convincing or consistent among all trials.

Accordingly, we reviewed all relevant studies and conducted a meta-analysis of RCTs to investigate the efficacy of acupuncture on hot flush and other symptoms of medical menopause in patients with breast cancer.

## Methods

### Data sources and searches

The conduct of this systematic review complied with the PRISMA Statement[19, 20] to ensure transparent and complete reporting. The following 7 databases were searched for relevant RCTs, with no language restrictions, from their inception dates to March 2017: MEDLINE (Ovid), EMBASE, Cochrane CENTRAL, CINAHL Plus with Full Text, Web of Science Core Collection, Index to Taiwan Periodical Literature System, and WHO International Clinical Trials Registry Platform (ICTRP). The reference lists of eligible articles were also reviewed to identify additional studies for possible inclusion. We also established e-mail alerts to identify newly released studies from the different databases that were within the scope of our review.

The keywords, hot flush, menopause symptoms, breast cancer, and acupuncture, used in the search included their synonyms (text words) and controlled vocabulary (MESH terms etc.) when available. Based on the MEDLINE (Ovid) search strategy, queries were revised for searches of the other databases. We adopted highly sensitive search syntaxes for identifying randomized trials. S1 Appendix shows the search strategy.

### Eligibility criteria

All eligible studies examined women with breast cancer, and measured the severity of hot flush and menopause symptoms. Only studies that used true needle acupuncture were enrolled, while other studies which adopted Transcutaneous electrical nerve stimulation (TENS) or other acupoints stimulation were excluded. To improve consistency, only studies that measured the frequency and severity of hot flush and the Kupperman index (a menopause rating scale)[21] were subjected to meta-analysis.

### Study and data extraction

**Fig 1:**
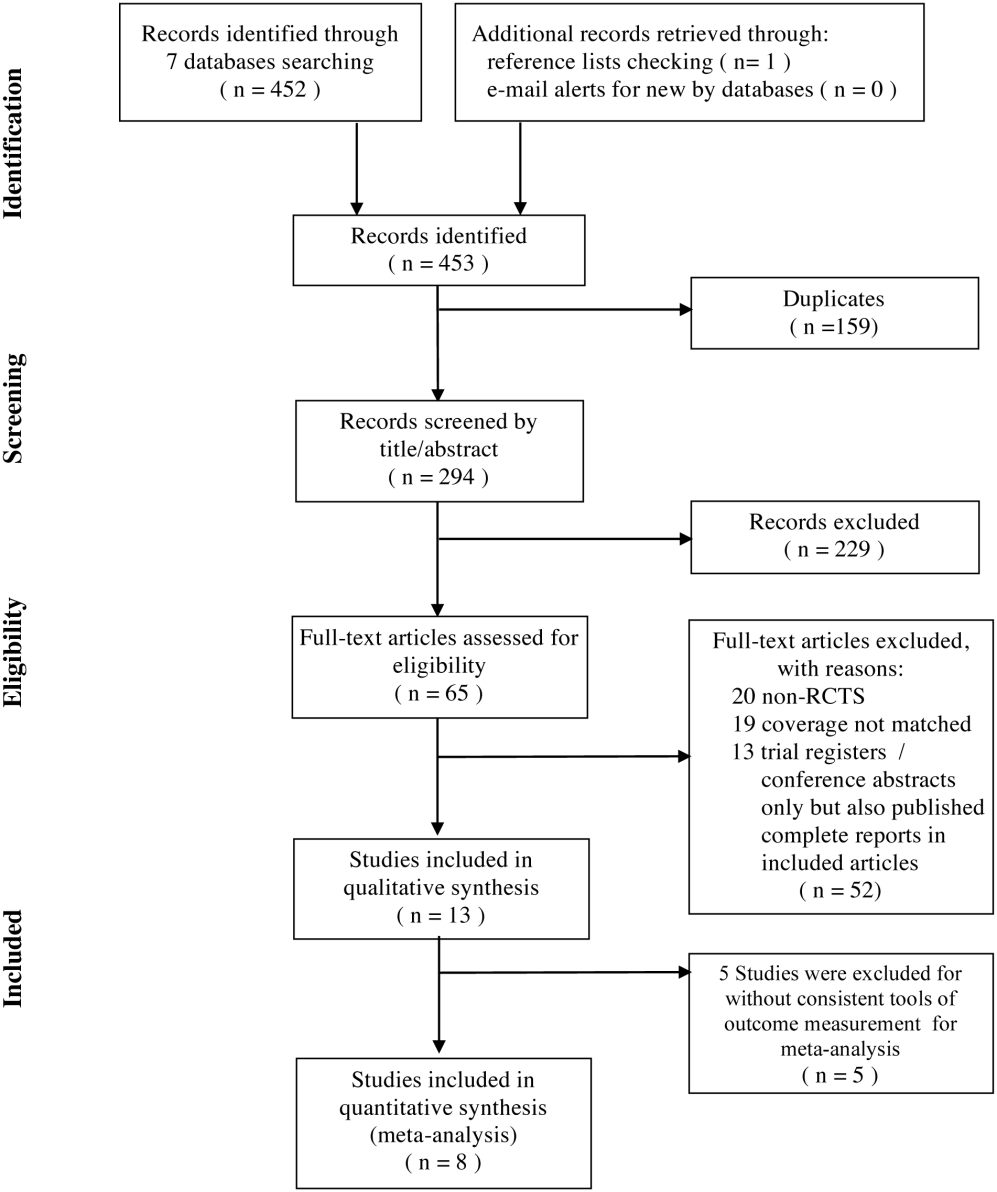
Selection of randomized controlled trials (based on PRISMA).

Searches of the 7 databases and additional sources led to the identification of 453 potentially relevant articles. The titles and abstracts that fulfilled the criteria of our study were independently read by two reviewers (Chien and Liu), and the full texts of articles that met these criteria were obtained. Final decisions on inclusion were made after examination of the full manuscripts. In cases of duplicate publications, the most recent and complete versions were selected. A total of 440 articles were excluded, 159 because they were duplicated, 229 that were considered irrelevant based on the titles and abstracts, and 52 full texts that were not RCTs, having non-matched coverage, or were trial registers / conference abstracts only were also excluded. We also contacted some authors to ensure that studies with only abstracts in some conferences yet have been published with completed data are included. (Fig 1).

There were 13 unique studies ultimately included. 7 studies compared acupuncture with sham acupuncture: Bao-2014[22], Hervik-2014[23], Bokmand-2013[24], Liljegren-2012[25], Hervik-2009[26], Deng-2007[27], Mao-2015[28]; 3 studies compared acupuncture with relaxation or self-care: Lesi-2016[29], Nedstrand-2006[30], Nedstrand-2005[31]; and 4 studies compared acupuncture with therapy by hormone or other medications: Frisk-2012[32], Frisk-2008[33], Walker-2010[34], and Mao-2015[28].

### The risk of bias and quality assessment

The quality of each enrolled study was assessed independently by 2 reviewers, using the criteria recommended in the *Cochrane Handbook for Systematic Reviews of Interventions*, *version 5*.*1*.*0*[35]. Six domains were assessed: *(i)* generation of randomization, *(ii)* allocation concealment, *(iii)* blinding of participants and personnel, *(iv)* blinding of outcome assessment, *(v)* incomplete outcome data, and *(vi)* selective outcome reporting. The modified Jadad scale was used to assess the quality of the included studies[36, 37]. Table 1 shows the risks of the bias in each of these 6 domains. We also contacted authors to identify additional studies and asked them for providing methodological details when data were missing.

**Table 1:** Risk of bias in the included randomized controlled trials.

Author^[ref]^, Year	Randomization	Allocation Concealment	Patients Blinding	Assessor Blinding	Incomplete Outcome Data Addressed	Selective Outcome Reporting	Modified Jadad Scale*
Lesi[29], 2016	Low	Low	Low	Low	Low	Low	5
Mao[28], 2015	Low	Low	Low	High	Low	Low	3
Bao[22], 2014	Low	Unclear	Low	Low	Low	Unclear	4
Hervik[23], 2014	Low	Low	Low	Low	Unclear	Unclear	4
Bokmand[24], 2013	Low	Low	Low	Low	Unclear	Low	4
Liljegren[25], 2012	Low	Low	Low	Low	Low	Unclear	5
Frisk[32], 2012	Low	Low	Unclear	Low	Low	Unclear	4
Walker[34], 2010	Low	Low	Low	Low	Unclear	Low	3
Hervik[26], 2009	Low	Low	Low	Low	Low	Low	5
Frisk[33], 2008	Low	Low	Low	Low	Low	Low	5
Deng[27], 2007	Low	Low	Low	Low	Low	Unclear	5
Nedstrand[30], 2006	Low	Low	Unclear	Low	Low	Unclear	4
Nedstrand[31], 2005	Low	Unclear	Unclear	Unclear	Low	Unclear	3

*Modified Jadad Scale: Jadad AR, Moore RA, Carroll D, Jenkinson C, Reynolds DJ, Gavaghan DJ, et al. Assessing the quality of reports of randomized clinical trials: is blinding necessary? Control Clin Trials. 1996;17(1):1–12.

### Data synthesis and statistical meta-analysis

To analyze the effects of acupuncture on outcomes (hot flush and menopausal symptoms), we estimated weighted mean differences (WMDs) and 95% confidence intervals (CIs) from each study using the Cochrane Collaboration software, Review Manager (RevMan) Version 5.2, for Windows. In estimating WMDs, a point estimate of zero indicated no effect, and an estimate less than zero indicated a benefit of acupuncture. Statistical heterogeneity was assessed using the Chi-square test (*p* < 0.1) and calculation of the I^2^ statistic. We considered an I^2^ value above 50% to indicate significant heterogeneity across studies[38]. A random-effects model was adapted if there was significant heterogeneity among studies. Otherwise, results were obtained from a fixed-effects model.

## Results

### Evaluation of quality and descriptions of the included trials

Table 1 shows the risk of bias in the 13 included studies. Most studies had low risk for adequate randomization, allocation, and blinding. The modified Jadad scale represents the quality of a RCT, and has a maximum of 5 points (1 point for randomization, 1 point for appropriate randomization method, 1 point for describing dropouts, 1 point for patient blinding, and 1 point for assessor blinding)[36]. The included studies had medium-to-high quality, in that 5 studies had scores of 5, 5 had scores of 4, and 3 had scores of 3.

The 13 prospective RCTs examined 844 patients with breast cancer (Table 2). The sample size ranged from 31 to 120, and the average age of enrolled subjects ranged from 50 to 61 years-old. The control treatments were sham acupuncture (7 studies), applied relaxation (3 studies), and hormone or drug therapy (4 studies). Some trials had more than one control group. The heterogeneity and limited number within individual enrolled articles might contribute to some risk of bias. Nevertheless, we only included eight studies with consistent outcomes for meta-analysis in order to reduce the possible bias. (Fig 1)

**Table 2:** Characteristics of the included randomized controlled trials.

Author^[ref]^, Year	Sample	Control Arm	Intervention (Primary Acupoints)	Course (Weeks)	Measurement Tools	Results
Lesi[29], 2016	105	Enhance self-care	10 acupuncture sessions once per week;SP6, LI11, CV4	12	Hot flush score Climacteric symptoms QoL	Acupuncture significantly decrease hot flush, climacteric syndrome and improve QoL
Mao[28], 2015	120	Sham acupuncture gabapentin	Twice per week for 2 weeks, then once per week for 6 more weeks,	8	Hot flush frequency Hot flush composite score (HFCS)	By week 8,SA produced significantly greater reduction in HFCS than did Placebo pills By week 24, HFCS reduction was greatest in the EA group, followed by Sham acupuncture, Placebo pill, and Gabapentin
Bao[22], 2014	47	Sham acupuncture	Weekly; CV4, CV6, CV12; LI4;MH6;GB34;ST36; KI3;BL65	8	NSABP; CESD; PSQI; HADS; Euro QoL; FRDI	SA group has significant change in flush frequency and severity; HFRDI, NSABP, Euro QoL
Hervik[23], 2014	61	Sham acupuncture	A course of 15 acupuncture treatments	10	Kupperman index	Acupuncture has positive effect on health related QoL
Bokmand[24], 2013	94	Sham acupuncture and no treatment	15–20 min weekly. HC6, KI3, SP6, LR3	12	VAS Estradiol level	Significantly relieve hot flush and sleep disturbance
Liljegren[25], 2012	84	Sham acupuncture	20 min twice a week for 5 weeks (De-Qi required); LI4, HT6, LR3, ST36, SP6, KI7	6	Frequency Severity score	No significant change in hot flush
Frisk[32], 2012	45	Hormone therapy	As standards for Reporting Interventions in Clinical Trials of Acupuncture	12	Hot flush scores PGWB WHO	Significant change of hot flush in both HT and EA group
Walker[34], 2010	50	SSRI: Venlafaxine	Twice-weekly for first 4 weeks, then weekly for 8 weeks. BL23, KI3, SP6, Du14,20, ST36, LI3, HE7.	12	Men-QoL SF12	Both groups: significant decrease hot flush, depression and QoL
Hervik[26], 2009	59	Sham acupuncture	30 min twice-weekly for 5 weeks then weekly for following 5 weeks. LIV3, GB20, LU7, KI3, SP6, REN4, P7, LIV8	10	Kupperman index	Significant improve in flush frequency
Frisk[33], 2008	45	Hormone therapy	30min twice-weekly for 2 weeks then weekly for 10 weeks	12	Flush frequency Kupperman index	Both groups noted significant change over flush frequency and depression
Deng[27], 2007	72	Sham acupuncture	Twice-weekly for 4 weeks. DU14, GB20, BL13, PC7, H6, K7, ST36, SP6	4	Flush frequency;	TA has longer benefit in reducing hot flush than SA
Nedstrand[30], 2006	31	Applied relaxation	30 min twice a week for 2 weeks then weekly for 10 weeks. (De Qi required)	12	VAS Mood scale	Both groups benefit from psychologic well-being; Mood improve in EA group
Nedstrand[31], 2005	31	Applied relaxation	30 min twice a week for 2 weeks then weekly for 10 weeks. (De Qi required) L15,23,32;HT7,SP6,9,LR3, PC6,GV20	12	Flush frequency Kupperman index	Both groups have significant change in flush frequency and KI

### Effect of acupuncture on the frequency and the severity of hot flush

**Fig 2:**
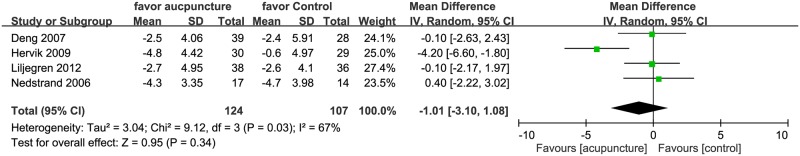
Forest plot of the effect of acupuncture on the frequency of hot flush. (times/day).

**Fig 3:**
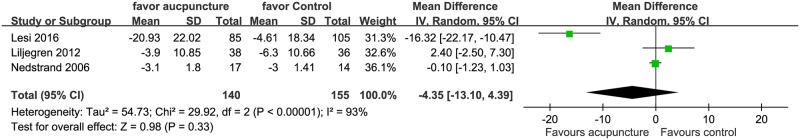
Forest plot of the effect of acupuncture on the severity of hot flush. (visual analog scale).

We observed significant between-study heterogeneity in the effects of acupuncture on the frequency of hot flush (times/day) (I2 = 67%), the severity of hot flush (visual analog scale) (I2 = 93%) and menopausal symptoms (menopause symptom scales) (I2 = 76%). Regarding the effect of acupuncture on the frequency of hot flush, total 124 patients were analyzed from 4 trials; no significant reduction in the frequency of hot flush was observed in subjects treated with acupuncture as compared with control subjects (-1.01 95% CI:-3.1, 1.08, *P* = 0.34; Fig 2). As for the 3 trials that reported data on severity of hot flush analyzed 140 patients; it indicated that acupuncture did not significantly reduce the severity of hot flush either. (Mean difference = -4.35, 95% CI:-13.10, 4.39, *P* = 0.33; Fig 3).

### Effect of acupuncture on menopausal symptoms

**Fig 4:**
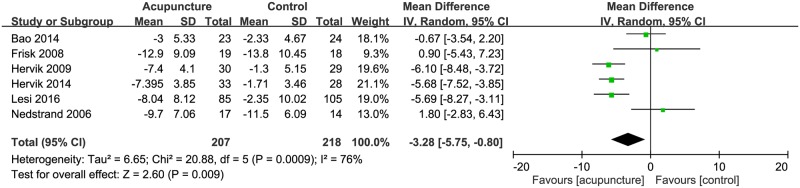
Forest plot of the effect of acupuncture on menopausal symptoms (scales).

Six trials (207 patients) reported data on menopausal symptoms, measured by menopause symptom scales (Fig 4). While most studies used the Kupperman index; Lesi (2016) adopted the Greene Climacteric scales, and Bao (2014) took NSABP menopausal symptom questionnaire; the different scales measure similar items without conflictions. To make sure the consistency, we transform the scores measured in Lesi and Bao’s study by proportion to match the scales of other studies. The results indicated that acupuncture significantly reduced menopausal symptoms (Mean difference = -3.28, 95% CI:-5.75, -0.80, *P* = 0.009; Fig 4).

Additionally, the funnel plot analysis is not eligible due to the insufficient number.

## Discussion

Most previous review studies that examined the effect of different treatments on hot flush examined hot flush frequency. We instead measured the effect of acupuncture on hot flush severity. Our results indicate that acupuncture had no significant effect on the severity of hot flush, but it did reduce general symptoms of menopause, as indicated by the Kupperman index. Below, we discuss studies with results inconsistent with the present meta-analysis, and the possible mechanism of acupuncture in relieving symptoms of menopause.

We included 3 studies that examined the effect of acupuncture on the severity of hot flush. Our meta-analysis indicated that acupuncture provided no significant improvement, leading us to ask why most individual studies showed a meaningful effect. It may be that although acupuncture had an effect, this effect was small, and because hormone therapy (the control therapy) had a similar effect in relieving hot flush, albeit with more adverse effects. The sham acupuncture may also have a positive, albeit small, effect in relieving the severity of hot flush.

Additionally, most studies used a visual analogue scale (VAS) or mood scale to evaluate the severity of hot flush. These scales may not be totally objective, because most women report an amelioration of hot flush during cold weather. The effect of seasonal changes as a possible bias in these studies should therefore be considered. Moreover, if acupuncture indeed significantly reduces the severity of hot flush, there must be some underlying mechanism. We have no evidence that acupuncture affects the level of estrogen, because most studies did not measure hormone levels. Nevertheless, some previous studies indicated no correlation between the plasma, urinary, or vaginal estrogen levels and the appearance of hot flush[39].

Aside from hot flush, factors such as negative mood, sensitivity to physical symptoms, sleep problems, longer duration of symptoms, and poorer health may contribute to an increased severity of vasomotor menopause syndrome. We therefore also investigated menopause symptoms, as indicated by the Kupperman index or other scales, in this meta-analysis. Our results indicated that acupuncture significantly ameliorated menopause symptoms. Most of the analyzed studies used the Kupperman index (with 21 questions), which evaluates hot flush, sleeping disorders, paresthesia, depression, joint pain, palpitation, headache, tingling, dizziness, and irritability. The results show that acupuncture provided a benefit (*p* = 0.009). We further examined the 2 studies that showed no effect^30, 33^. The Nedstrand et al. study reported the control and acupuncture groups both had significant amelioration of climacteric symptoms[30]. Similarly, the Frisk et al. study, which compared electro-acupuncture to hormone therapy, reported a benefit from both treatments, but that hormone therapy had a stronger effect[33].

Studies of the mechanism of hot flush indicate that estrogen depletion has an important role. Hot flush occurs in most women undergoing natural or medical menopause, and is related to cessation of ovary function[40, 41]. However, estrogen depletion is not the sole factor, because there are no differences in plasma estrogen levels between symptomatic and asymptomatic women[42, 43]. Recent studies suggest that hot flush might be related to autonomic dysfunction[44, 45], and disruption of thermoregulation in the hypothalamus, leading to increased core body temperature (Tc)[43, 46]. Other research suggested that the rate of change in plasma estrogen concentration influences the thermoregulatory system *via* the hypothalamus[47].

These intriguing previous studies lead us to ask: “How can acupuncture alleviate hot flush?” and “By what mechanism does acupuncture relieve menopause symptoms?” There is some evidence that acupuncture may relieve menopause symptoms and hot flush *via* altering the autonomic nervous system. Moreover, the thermo-neutral zone of women undergoing menopause may be narrowed due to elevated sympathetic activity, thus resulting in hot flush and elevation of core body temperature (Tc)[48]. Freedman also noted that up-regulation of the sympathetic system is an essential component of hot flush[49]. Hence, acupuncture might up-regulate the parasympathetic system, counter-balancing the over-excited sympathetic system, and thereby relieve menopause symptoms.

From the perspective of neurotransmitters, some evidence suggests that the decrease of estrogen correlates with the decline of endorphins in the hypothalamus. A reduced level of endorphins promotes the release of serotonin and norepinephrine, which contribute to the decline in the set-point in the thermoregulatory center of the hypothalamus, and triggers hot flush and vasomotor menopause symptoms[50, 51]. It is widely acknowledged that acupuncture can stimulate the secretion of endorphins[52] and alter autonomic nerve function[53, 54]. This mechanism may therefore explain how acupuncture alleviates menopause symptoms, such as joint pain, sleeping disorder, depression, and nervous system-related symptoms such as paresthesia and tingling[55].

Recently, some scholars proposed that Neurokinin B (NKB) administration can cause hot flushes in women as it is a hypothalamic neuropeptide binding to the neurokinin 3 receptors.[56]. Similar research also support that the marked changes in hypothalamic kisspeptin, neurokinin B and dynorphin (KNDy) neurons might contribute to hot flush based on the animal study noted that KNDy neurons modulate LH secretion and body temperature[57]. These statements remind us the acupuncture could probably improve hot flush by regulating these transmitters though there are still need more solid research[58].

The mechanism by which acupuncture alleviates menopause syndrome might be complex, and involve more than simply regulating cytokines and the autonomic nerve systems, yet our interpretation is reasonable and consistent with available research. Nonetheless, we cannot draw a definitive conclusion, because an insufficient number of acupuncture studies have examined biomarkers or cytokines. Future studies should focus on the underlying molecular mechanisms of acupuncture, and should attempt to reinterpret the traditional theory of meridians and qi, and identify therapeutic indications within the framework of evidence-based medicine.

## Limitations

This meta-analysis only examined RCTs to achieve higher internal validity, but there were still some limitations. First, the number of the enrolled patients in each study was small (range: 31 to 120). Second, variations among the included studies, such as use of different controls, treatment sessions and periods, acupoints, and menopause status of patients, may have led to bias. Third, some studies did not clearly state whether patients and assessors were blinded, and some did not address the presence of incomplete outcome data or whether there was selective outcome reporting. Despite these limitations, none of the studies reported severe adverse effects, although there were some minor events, such as slight bleeding and/or bruising at the site of needle insertion.

## Conclusion

This meta-analysis of women with breast cancer confirms that acupuncture had no significant effect on the severity of hot flush, but did significantly alleviate the symptoms of menopause. We conclude that acupuncture is non-inferior to hormone therapy or other applied relaxation therapies in alleviating the symptoms of menopause for its safety, non-invasive and especially considering the side effect of hormone therapy. Recent studies show that use of acupuncture does not correlate with increased levels of plasma estradiol. Considering the safety and lack of serious adverse effects associated with acupuncture, in contrast to hormone therapy, acupuncture should be considered for treating hot flush and menopause syndrome in women with breast cancer.

## Supporting information

S1 Appendix Search strategy. (DOCX)

S2 Appendix PRISMA 2009 checklist. (DOC)

## References

[1] Otte JL, Carpenter JS, Zhong X, Johnstone PA. Feasibility study of acupuncture for reducing sleep disturbances and hot flush in postmenopausal breast cancer survivors. Clin Nurse Spec. 2011;25(5):228–36. doi: 10.1097/NUR.0b013e318229950b 22366695 10.1097/NUR.0b013e318229950b PMC3292187

[2] Otte JL, Carpenter JS, Russell KM, Bigatti S, Champion VL. Prevalence, severity, and correlates of sleep-wake disturbances in long-term breast cancer survivors. J Pain Symptom Manage. 2010;39(3):535–47. doi: 10.1016/j.jpainsymman.2009.07.004 20083371 10.1016/j.jpainsymman.2009.07.004 PMC2843803

[3] Desai K, Mao JJ, Su I, Demichele A, Li Q, Xie SX, et al Prevalence and risk factors for insomnia among breast cancer patients on aromatase inhibitors. Support Care Cancer. 2013;21(1):43–51. doi: 10.1007/s00520-012-1490-z 22584732 10.1007/s00520-012-1490-z PMC3600410

[4] Seib C, Anderson D, Lee K. Prevalence and correlates of sleep disturbance in postmenopausal women: the Australian Healthy Aging of Women (HOW) Study. J Womens Health (Larchmt). 2014;23(2):151–8. doi: 10.1089/jwh.2013.4472 24261649 10.1089/jwh.2013.4472

[5] Couzi RJ, Helzlsouer KJ, Fetting JH. Prevalence of menopausal symptoms among women with a history of breast cancer and attitudes toward estrogen replacement therapy. J Clin Oncol. 1995;13(11):2737–44. doi: 10.1200/JCO.1995.13.11.2737 7595732 10.1200/JCO.1995.13.11.2737

[6] Johns C, Seav SM, Dominick SA, Gorman JR, Li HY, Natarajan L, et al Informing hot flash treatment decisions for breast cancer survivors: a systematic review of randomized trials comparing active interventions. Breast Cancer Res Treat. 2016;156(3):415–26. doi: 10.1007/s10549-016-3765-4 27015968 10.1007/s10549-016-3765-4 PMC4838539

[7] Holmberg L, Iversen OE, Rudenstam CM, Hammar M, Kumpulainen E, Jaskiewicz J, et al Increased risk of recurrence after hormone replacement therapy in breast cancer survivors. J Natl Cancer Inst. 2008;100(7):475–82. doi: 10.1093/jnci/djn058 18364505 10.1093/jnci/djn058

[8] Schenck-Gustafsson K, Brincat M, Erel CT, Gambacciani M, Lambrinoudaki I, Moen MH, et al EMAS position statement: managing the menopause in the context of coronary heart disease. Maturitas. 2011;68(1):94–7. doi: 10.1016/j.maturitas.2010.10.005 21156341 10.1016/j.maturitas.2010.10.005

[9] Albertazzi P. A review of non-hormonal options for the relief of menopausal symptoms. Treat Endocrinol. 2006;5(2):101–13. 16542050 10.2165/00024677-200605020-00004

[10] Philp HA. Hot flashes—a review of the literature on alternative and complementary treatment approaches. Altern Med Rev. 2003;8(3):284–302. 12946239

[11] Selva Olid A, Martinez Zapata MJ, Sola I, Stojanovic Z, Uriona Tuma SM, Bonfill Cosp X. Efficacy and safety of needle acupuncture for treating gynecologic and obstetric disorders: an overview. Med Acupunct. 2013;25(6):386–97. doi: 10.1089/acu.2013.0976 24761184 10.1089/acu.2013.0976 PMC3870573

[12] Joshi N, Araque H. Neurophysiologic basis for the relief of human pain by acupuncture. Acupunct Electrother Res. 2009;34(3–4):165–74. 20344884 10.3727/036012909803861022

[13] Filshie J, Bolton T, Browne D, Ashley S. Acupuncture and self acupuncture for long-term treatment of vasomotor symptoms in cancer patients—audit and treatment algorithm. Acupunct Med. 2005;23(4):171–80. 16430125 10.1136/aim.23.4.171

[14] Sturdee DW. The menopausal hot flush—anything new? Maturitas. 2008;60(1):42–9. doi: 10.1016/j.maturitas.2008.02.006 18384981 10.1016/j.maturitas.2008.02.006

[15] Wanchai A, Armer JM, Stewart BR. Complementary and alternative medicine use among women with breast cancer: a systematic review. Clin J Oncol Nurs. 2010;14(4):E45–55. doi: 10.1188/10.CJON.E45-E55 20682492 10.1188/10.CJON.E45-E55

[16] Schapira MM, Mackenzie ER, Lam R, Casarett D, Seluzicki CM, Barg FK, et al Breast cancer survivors willingness to participate in an acupuncture clinical trial: a qualitative study. Support Care Cancer. 2014;22(5):1207–15. doi: 10.1007/s00520-013-2073-3 24362843 10.1007/s00520-013-2073-3 PMC4162629

[17] Chiu HY, Hsieh YJ, Tsai PS. Acupuncture to reduce sleep disturbances in perimenopausal and postmenopausal women: a systematic review and meta-analysis. Obstet Gynecol. 2016;127(3):507–15. doi: 10.1097/AOG.0000000000001268 26855097 10.1097/AOG.0000000000001268

[18] Cho SH, Whang WW. Acupuncture for vasomotor menopausal symptoms: a systematic review. Menopause. 2009;16(5):1065–73. doi: 10.1097/gme.0b013e3181a48abd 19424092 10.1097/gme.0b013e3181a48abd

[19] Moher D, Liberati A, Tetzlaff J, Altman DG, Group P. Preferred reporting items for systematic reviews and meta-analyses: the PRISMA statement. PLoS Med. 2009;6(7):e1000097 doi: 10.1371/journal.pmed.1000097 19621072 10.1371/journal.pmed.1000097 PMC2707599

[20] Liberati A, Altman DG, Tetzlaff J, Mulrow C, Gotzsche PC, Ioannidis JP, et al The PRISMA statement for reporting systematic reviews and meta-analyses of studies that evaluate health care interventions: explanation and elaboration. PLoS Med. 2009;6(7):e1000100 doi: 10.1371/journal.pmed.1000100 19621070 10.1371/journal.pmed.1000100 PMC2707010

[21] Schneider HP, Heinemann LA, Rosemeier HP, Potthoff P, Behre HM. The Menopause Rating Scale (MRS): comparison with Kupperman index and quality-of-life scale SF-36. Climacteric. 2000;3(1):50–8. 11910610 10.3109/13697130009167599

[22] Bao T, Cai L, Snyder C, Betts K, Tarpinian K, Gould J, et al Patient-reported outcomes in women with breast cancer enrolled in a dual-center, double-blind, randomized controlled trial assessing the effect of acupuncture in reducing aromatase inhibitor-induced musculoskeletal symptoms. Cancer. 2014;120(3):381–9. doi: 10.1002/cncr.28352 24375332 10.1002/cncr.28352 PMC3946917

[23] Hervik J, Mjaland O. Long term follow up of breast cancer patients treated with acupuncture for hot flashes. Springerplus. 2014;3:141 doi: 10.1186/2193-1801-3-141 25674442 10.1186/2193-1801-3-141 PMC4320138

[24] Bokmand S, Flyger H. Acupuncture relieves menopausal discomfort in breast cancer patients: a prospective, double blinded, randomized study. Breast. 2013;22(3):320–3. doi: 10.1016/j.breast.2012.07.015 22906948 10.1016/j.breast.2012.07.015

[25] Mao JJ, Xie SX, Bowman MA, Bruner D, Li SQ, DeMichele A, et al A randomized placebo-controlled trial of acupuncture and gabapentin for hot flashes among breast cancer survivors. Cancer Res. 2015;75(9 suppl. 1). doi: 10.1158/1538-7445.SABCS14-PD4-7 CN-01084687 10.1200/JCO.2015.60.9412 PMC4622101 26304905

[26] Hervik J, Mjaland O. Acupuncture for the treatment of hot flashes in breast cancer patients, a randomized, controlled trial. Breast Cancer Res Treat. 2009;116(2):311–6. doi: 10.1007/s10549-008-0210-3 18839306 10.1007/s10549-008-0210-3

[27] Deng G, Vickers AJ, Yeung KS, D'Andrea GM, Xiao H, Heerdt AS, et al Randomized, controlled trial of acupuncture for the treatment of hot flashes in breast cancer patients. J Clin Oncol. 2007;25(35):5584–90. doi: 10.1200/JCO.2007.12.0774 18065731 10.1200/JCO.2007.12.0774

[28] Mao JJ, Bowman MA, Xie SX, Bruner D, DeMichele A, Farrar JT. Electroacupuncture versus gabapentin for hot flashes among breast cancer survivors: a randomized placebo-controlled trial. J Clin Oncol. 2015;33(31):3615–20. Epub 2015/08/26. doi: 10.1200/JCO.2015.60.9412 26304905 10.1200/JCO.2015.60.9412 PMC4622101

[29] Lesi G, Razzini G, Musti MA, Stivanello E, Petrucci C, Benedetti B, et al Acupuncture ss an integrative approach for the treatment of hot flashes in women with breast cancer: a prospective multicenter randomized controlled trial (AcCliMaT). J Clin Oncol. 2016;34(15):1795–802. doi: 10.1200/JCO.2015.63.2893 27022113 10.1200/JCO.2015.63.2893

[30] Nedstrand E, Wyon Y, Hammar M, Wijma K. Psychological well-being improves in women with breast cancer after treatment with applied relaxation or electro-acupuncture for vasomotor symptom. J Psychosom Obstet Gynaecol. 2006;27(4):193–9. 17225620 10.1080/01674820600724797

[31] Nedstrand E, Wijma K, Wyon Y, Hammar M. Vasomotor symptoms decrease in women with breast cancer randomized to treatment with applied relaxation or electro-acupuncture: a preliminary study. Climacteric. 2005;8(3):243–50. doi: 10.1080/13697130500118050 16390756 10.1080/13697130500118050

[32] Frisk J, Kallstrom AC, Wall N, Fredrikson M, Hammar M. Acupuncture improves health-related quality-of-life (HRQoL) and sleep in women with breast cancer and hot flushes. Support Care Cancer. 2012;20(4):715–24. doi: 10.1007/s00520-011-1134-8 21468626 10.1007/s00520-011-1134-8

[33] Frisk J, Carlhall S, Kallstrom AC, Lindh-Astrand L, Malmstrom A, Hammar M. Long-term follow-up of acupuncture and hormone therapy on hot flushes in women with breast cancer: a prospective, randomized, controlled multicenter trial. Climacteric. 2008;11(2):166–74. doi: 10.1080/13697130801958709 18365859 10.1080/13697130801958709

[34] Walker EM, Rodriguez AI, Kohn B, Ball RM, Pegg J, Pocock JR, et al Acupuncture versus venlafaxine for the management of vasomotor symptoms in patients with hormone receptor-positive breast cancer: a randomized controlled trial. J Clin Oncol. 2010;28(4):634–40. doi: 10.1200/JCO.2009.23.5150 20038728 10.1200/JCO.2009.23.5150

[35] Higgins JPT, Green S. Cochrane Handbook for Systematic Reviews of Interventions version 5.1.0 20 March 2011. http://handbook.cochrane.org/. Cited 10 March 2017.

[36] Jadad AR, Moore RA, Carroll D, Jenkinson C, Reynolds DJ, Gavaghan DJ, et al Assessing the quality of reports of randomized clinical trials: is blinding necessary? Control Clin Trials. 1996;17(1):1–12. 8721797 10.1016/0197-2456(95)00134-4

[37] Gao Z, Liu Y, Zhang J, Upur H. Effect of Jianpi therapy in treatment of chronic obstructive pulmonary disease: a systematic review. J Tradit Chin Med. 2013;33(1):1–8. 23596804 10.1016/s0254-6272(13)60092-8

[38] Higgins JP, Thompson SG, Deeks JJ, Altman DG. Measuring inconsistency in meta-analyses. BMJ. 2003;327(7414):557–60. doi: 10.1136/bmj.327.7414.557 12958120 10.1136/bmj.327.7414.557 PMC192859

[39] Freedman RR. Physiology of hot flashes. Am J Hum Biol. 2001;13(4):453–64. doi: 10.1002/ajhb.1077 11400216 10.1002/ajhb.1077

[40] Thurston RC, Bromberger JT, Joffe H, Avis NE, Hess R, Crandall CJ, et al Beyond frequency: who is most bothered by vasomotor symptoms? Menopause. 2008;15(5):841–7. doi: 10.1097/gme.0b013e318168f09b 18521049 10.1097/gme.0b013e318168f09b PMC2866103

[41] Morrow PKH, Mattair DN, Hortobagyi GN. Hot flashes: a review of pathophysiology and treatment modalities. Oncologist. 2011;16(11):1658–64. doi: 10.1634/theoncologist.2011-0174 22042786 10.1634/theoncologist.2011-0174 PMC3233302

[42] Aksel S, Schomberg DW, Tyrey L, Hammond CB. Vasomotor symptoms, serum estrogens, and gonadotropin levels in surgical menopause. Am J Obstet Gynecol. 1976;126(2):165–9. 961757 10.1016/0002-9378(76)90270-2

[43] Freedman RR, Norton D, Woodward S, Cornelissen G. Core body temperature and circadian rhythm of hot flashes in menopausal women. J Clin Endocrinol Metab. 1995;80(8):2354–8. doi: 10.1210/jcem.80.8.7629229 7629229 10.1210/jcem.80.8.7629229

[44] Conti FF, Brito Jde O, Bernardes N, Dias Dda S, Sanches IC, Malfitano C, et al Cardiovascular autonomic dysfunction and oxidative stress induced by fructose overload in an experimental model of hypertension and menopause. BMC Cardiovasc Disord. 2014;14:185 doi: 10.1186/1471-2261-14-185 25495455 10.1186/1471-2261-14-185 PMC4279597

[45] Gorodeski EZ. Autonomic dysfunction: a common mechanism for heart failure and hot flashes? Menopause. 2012;19(4):382–3. doi: 10.1097/gme.0b013e31824c7a3b 22453130 10.1097/gme.0b013e31824c7a3b

[46] Freedman RR. Menopausal hot flashes: mechanisms, endocrinology, treatment. J Steroid Biochem Mol Biol. 2014;142:115–20. doi: 10.1016/j.jsbmb.2013.08.010 24012626 10.1016/j.jsbmb.2013.08.010 PMC4612529

[47] Andrikoula M, Prelevic G. Menopausal hot flushes revisited. Climacteric. 2009;12(1):3–15. doi: 10.1080/13697130802556296 19061056 10.1080/13697130802556296

[48] Freedman RR. Pathophysiology and treatment of menopausal hot flashes. Semin Reprod Med. 2005;23(2):117–25. doi: 10.1055/s-2005-869479 15852197 10.1055/s-2005-869479

[49] Freedman RR. Menopausal hot flashes In: Lobo RA, editor. Treatment of the Postmenopausal Woman: Basic and Clinical Aspects. 3rd ed Burlington, MA: Academic Press; 2007 pp. 187–98.

[50] Borud E, White A. A review of acupuncture for menopausal problems. Maturitas. 2010;66(2):131–4. doi: 10.1016/j.maturitas.2009.12.010 20060667 10.1016/j.maturitas.2009.12.010

[51] Freedman RR, Krell W. Reduced thermoregulatory null zone in postmenopausal women with hot flashes. Am J Obstet Gynecol. 1999;181(1):66–70. 10411797 10.1016/s0002-9378(99)70437-0

[52] Andersson S, Lundeberg T. Acupuncture—from empiricism to science: functional background to acupuncture effects in pain and disease. Med Hypotheses. 1995;45(3):271–81. 8569551 10.1016/0306-9877(95)90117-5

[53] Wang JD, Kuo TB, Yang CC. An alternative method to enhance vagal activities and suppress sympathetic activities in humans. Auton Neurosci. 2002;100(1–2):90–5. 12422965 10.1016/s1566-0702(02)00150-9

[54] Loaiza LA, Yamaguchi S, Ito M, Ohshima N. Electro-acupuncture stimulation to muscle afferents in anesthetized rats modulates the blood flow to the knee joint through autonomic reflexes and nitric oxide. Auton Neurosci. 2002;97(2):103–9. 12132642 10.1016/s1566-0702(02)00051-6

[55] Borud E, Grimsgaard S, White A. Menopausal problems and acupuncture. Autonomic Neuroscience-Basic & Clinical. 2010;157(1–2):57–62. doi: 10.1016/j.autneu.2010.04.004 20447875 10.1016/j.autneu.2010.04.004

[56] Jayasena CN, Comninos AN, Stefanopoulou E, Buckley A, Narayanaswamy S, Izzi-Engbeaya C, et al Neurokinin B administration induces hot flushes in women. Sci Rep. 2015;5:8466 doi: 10.1038/srep08466 25683060 10.1038/srep08466 PMC4329553

[57] Rance NE, Dacks PA, Mittelman-Smith MA, Romanovsky AA, Krajewski-Hall SJ. Modulation of body temperature and LH secretion by hypothalamic KNDy (kisspeptin, neurokinin B and dynorphin) neurons: a novel hypothesis on the mechanism of hot flushes. Front Neuroendocrinol. 2013;34(3):211–27. doi: 10.1016/j.yfrne.2013.07.003 23872331 10.1016/j.yfrne.2013.07.003 PMC3833827

[58] Zhang W, Jiao L, Xiong J. [Effects of Suspending Moxibustion at "Dazhui" (GV 14) on Neurogenic Inflammation in Asthma Rats]. Zhen Ci Yan Jiu. 2015;40(5):388–91. 26669196

