# Supplementary material for: Effect of acupuncture on hot flush and menopause symptoms in breast cancer- A systematic review and meta-analysis
Source: PLoS One. 2017 Aug 22;12(8):e0180918. doi: 10.1371/journal.pone.0180918 (PMC5568723; doi:10.1371/journal.pone.0180918)
Supplement: S1 Appendix — (DOCX) [file pone.0180918.s001.docx]

**S1 Appendix. Search Strategy**

| **Database** | **#** | **Search syntax** | **Citations found** |
| --- | --- | --- | --- |
| **MEDLINE** | 1 | exp Hot Flashes/ OR exp Vasomotor System/ OR exp Sweating/ |  |
|  | 2 | (flush* OR ((hot OR night* OR nocturnal*) ADJ5 (flash* OR sweat*)) OR vasomotor).mp |  |
|  | 3 | exp Climacteric/ OR exp Primary Ovarian Insufficiency/ OR exp Anovulation/ |  |
|  | 4 | (climacter* OR menopaus* OR premenopaus* OR perimenopaus* OR postmenopaus* OR pre-menopaus* OR peri-menopaus* OR post-menopaus* OR (mens ADJ3 cessat*) OR (ovarian ADJ3 (fail* OR cessat* OR absen*)) OR anovulation*).mp |  |
|  | 5 | exp Breast Neoplasms/ |  |
|  | 6 | ((breast OR mamma*) ADJ11 (cancer* OR neopla* OR adenocarcin* OR carcin* OR tumor* OR tumour* OR malignan* OR sarcoma* OR mass* OR DCIS OR ductal* OR infiltrat* OR intraductal* OR lobula* OR medullary)).mp |  |
|  | 7 | exp Acupuncture/ OR exp Acupuncture Therapy/ OR exp Acupressure/ OR exp Transcutaneous Electric Nerve Stimulation/ |  |
|  | 8 | (acupunctur* OR acupress* OR acupoint* OR electroacupunctur* OR Shiatsu OR Shiatzu OR "Zhi Ya" OR ZhiYa OR "Chih Ya" OR ChihYa OR "Zhen Jiu" OR ZhenJiu OR "Tui Na" OR TuiNa OR meridian* OR "Ching Lo" OR Chinglo OR "Jing Luo" OR Jingluo OR moxibustion* OR auriculotherapy OR TENS OR PENS OR ((transcutaneous OR percutaneous OR transdermal OR cutaneous) ADJ4 (stimulat* OR electrostimulat*))).mp |  |
|  | 9 | (1 OR 2 OR 3 OR 4) AND (5 OR 6) AND (7 OR 8) | 101 |
|  | 10 | 9 and ((randomized controlled trial.pt. or controlled clinical trial.pt. or randomi?ed.ab. or placebo.ab. or drug therapy.fs. or randomly.ab. or trial.ab. or groups.ab.) not (exp animals/ not humans.sh.)) | **RCTs: 74** |
| **EMBASE** | 1 | 'hot flush'/exp OR 'vasomotor disorder'/exp OR 'night sweat'/exp |  |
|  | 2 | (flush* OR ((hot OR night* OR nocturnal*) NEAR/4 (flash* OR sweat*)) OR vasomotor):ti,ab,de |  |
|  | 3 | 'menopause and climacterium'/exp OR **'**menopause related disorder'/de OR 'menopausal syndrome'/exp OR 'premature ovarian failure'/exp OR 'Anovulation'/exp |  |
|  | 4 | (climacter* OR menopaus* OR premenopaus* OR perimenopaus* OR postmenopaus* OR pre-menopaus* OR peri-menopaus* OR post-menopaus* OR (mens NEAR/2 cessat*) OR (ovarian NEAR/2 (fail* OR cessat* OR absen*)) OR anovulation*):ti,ab,de |  |
|  | 5 | 'breast tumor'/exp |  |
|  | 6 | ((breast OR mamma*) NEAR/10 (cancer* OR neopla* OR adenocarcin* OR carcin* OR tumor* OR tumour* OR malignan* OR sarcoma* OR mass* OR DCIS OR ductal* OR infiltrat* OR intraductal* OR lobula* OR medullary)):ti,ab,de |  |
|  | 7 | 'acupuncture'/exp OR 'transcutaneous nerve stimulation'/exp OR 'moxibustion'/exp |  |
|  | 8 | (acupunctur* OR acupress* OR acupoint* OR electroacupunctur* OR Shiatsu OR Shiatzu OR "Zhi Ya" OR ZhiYa OR "Chih Ya" OR ChihYa OR "Zhen Jiu" OR ZhenJiu OR "Tui Na" OR TuiNa OR meridian* OR "Ching Lo" OR Chinglo OR "Jing Luo" OR Jingluo OR moxibustion* OR auriculotherapy OR TENS OR PENS OR ((transcutaneous OR percutaneous OR transdermal OR cutaneous) NEAR/3 (stimulat* OR electrostimulat*))):ti,ab,de |  |
|  | 9 | (#1 OR #2 OR #3 OR #4) AND (#5 OR #6) AND (#7 OR #8) | 330 |
|  | 10 | #9 AND ('crossover procedure':de OR 'double-blind procedure':de OR 'randomized controlled trial':de OR  'single-blind procedure':de OR (random* OR  factorial* OR crossover* OR cross NEXT/1 over* OR placebo* OR doubl* NEAR/1 blind* OR singl* NEAR/1 blind* OR assign* OR allocat* OR volunteer*):de,ab,ti) | **RCTs: 113** |
| **Cochrane**  **CENTRAL** | 1 | MeSH descriptor: [Hot Flashes] explode all trees |  |
|  | 2 | MeSH descriptor: [Vasomotor System] explode all trees |  |
|  | 3 | MeSH descriptor: [Sweating] explode all trees |  |
|  | 4 | (flush* or ((hot or night* or nocturnal*) near/4 (flash* or sweat*)) or vasomotor):ti,ab,kw |  |
|  | 5 | MeSH descriptor: [Climacteric] explode all trees |  |
|  | 6 | MeSH descriptor: [Primary Ovarian Insufficiency] explode all trees |  |
|  | 7 | MeSH descriptor: [Anovulation] explode all trees |  |
|  | 8 | (climacter* OR menopaus* OR premenopaus* OR perimenopaus* OR postmenopaus* OR pre-menopaus* OR peri-menopaus* OR post-menopaus* OR (mens NEAR2 cessat*) OR (ovarian NEAR2 (fail* OR cessat* OR absen*)) OR anovulation*).ti,ab,kw |  |
|  | 9 | MeSH descriptor: [Breast Neoplasms] explode all trees |  |
|  | 10 | ((breast OR mamma*) NEAR10 (cancer* OR neopla* OR adenocarcin* OR carcin* OR tumor* OR tumour* OR malignan* OR sarcoma* OR mass* OR DCIS OR ductal* OR infiltrat* OR intraductal* OR lobula* OR medullary)):ti,ab,kw |  |
|  | 11 | MeSH descriptor: [Acupuncture] explode all trees |  |
|  | 12 | MeSH descriptor: [Acupuncture Therapy] explode all trees |  |
|  | 13 | MeSH descriptor: [Acupressure] explode all trees |  |
|  | 14 | MeSH descriptor: [Transcutaneous Electric Nerve Stimulation] explode all trees |  |
|  | 15 | (acupunctur* OR acupress* OR acupoint* OR electroacupunctur* OR Shiatsu OR Shiatzu OR "Zhi Ya" OR ZhiYa OR "Chih Ya" OR ChihYa OR "Zhen Jiu" OR ZhenJiu OR "Tui Na" OR TuiNa OR meridian* OR "Ching Lo" OR Chinglo OR "Jing Luo" OR Jingluo OR moxibustion* OR auriculotherapy OR TENS OR PENS OR ((transcutaneous OR percutaneous OR transdermal OR cutaneous) NEAR3 (stimulat* OR electrostimulat*))):ti,ab,kw |  |
|  | 16 | (#1 or #2 or #3 or #4 or #5 or #6 or #7 or #8) and (#9 or #10) and (#11 or #12 or #14 or #15) | **CTs: 51** |
| **CINAHL Plus with Full Text** | 1 | (MH "Hot Flashes+") OR (MH "Sweating+") |  |
|  | 2 | (flush* OR ((hot OR night* OR nocturnal*) N4 (flash* OR sweat*)) OR vasomotor) |  |
|  | 3 | (MH "Climacteric+") OR (MH "Anovulation+") |  |
|  | 4 | (climacter* OR menopaus* OR premenopaus* OR perimenopaus* OR postmenopaus* OR pre-menopaus* OR peri-menopaus* OR post-menopaus* OR (mens N2 cessat*) OR (ovarian N2 (fail* OR cessat* OR absen*)) OR anovulation*) |  |
|  | 5 | (MH "Breast Neoplasms+") |  |
|  | 6 | ((breast OR mamma*) N10 (cancer* OR neopla* OR adenocarcin* OR carcin* OR tumor* OR tumour* OR malignan* OR sarcoma* OR mass* OR DCIS OR ductal* OR infiltrat* OR intraductal* OR lobula* OR medullary)) |  |
|  | 7 | (MH "Acupuncture+") OR (MH "Transcutaneous Electric Nerve Stimulation+") |  |
|  | 8 | acupunctur* OR acupress* OR acupoint* OR electroacupunctur* OR Shiatsu OR Shiatzu OR "Zhi Ya" OR ZhiYa OR "Chih Ya" OR ChihYa OR "Zhen Jiu" OR ZhenJiu OR "Tui Na" OR TuiNa OR meridian* OR "Ching Lo" OR Chinglo OR "Jing Luo" OR Jingluo OR moxibustion* OR auriculotherapy OR "TENS" OR PENS OR ((transcutaneous OR percutaneous OR transdermal OR cutaneous) N3 (stimulat* OR electrostimulat*)) |  |
|  | 9 | (s1 OR s2 OR s3 OR s4) AND (s5 OR s6) AND (s7 OR s8) | 92 |
|  | 10 | s9 and ((MH "Clinical Trials+") or (PT Clinical trial) or (TX clinic* n1 trial*) or TX ( (singl* n1 blind*) or (singl* n1 mask*) ) or TX ( (doubl* n1 blind*) or (doubl* n1 mask*) ) or TX ( (tripl* n1 blind*) or (tripl* n1 mask*) ) or TX ( (trebl* n1 blind*) or (trebl* n1 mask*) ) or (TX randomi* control* trial*) or (MH "Random Assignment") or (TX random* allocat*) or (TX placebo*) or (MH "Placebos") or (MH "Placebos") or (MH "Quantitative Studies") or (TX allocat* random*)) | **RCTs: 56** |
| **Web of Scienceo Core Collection** | 1 | (flush* OR ((hot OR night* OR nocturnal*) near/4 (flash* OR sweat*)) OR vasomotor) |  |
|  | 2 | (climacter* OR *menopaus* OR (mens near/2 cessat*) OR (ovarian near/2 (fail* OR cessat* OR absen*)) OR anovulation*) |  |
|  | 3 | ((breast OR mamma*) near/10 (cancer* OR neopla* OR adenocarcin* OR carcin* OR tumor* OR tumour* OR malignan* OR sarcoma* OR mass* OR DCIS OR ductal* OR infiltrat* OR intraductal* OR lobula* OR medullary)) |  |
|  | 4 | (acupunctur* OR acupress* OR acupoint* OR electroacupunctur* OR Shiatsu OR Shiatzu OR "Zhi Ya" OR ZhiYa OR "Chih Ya" OR ChihYa OR "Zhen Jiu" OR ZhenJiu OR "Tui Na" OR TuiNa OR meridian* OR "Ching Lo" OR Chinglo OR "Jing Luo" OR Jingluo OR moxibustion* OR auriculotherapy OR TENS OR PENS OR ((transcutaneous OR percutaneous OR transdermal OR cutaneous) near/3 (stimulat* OR electrostimulat*))) |  |
|  | 5 | (#1 OR #2) AND #3 AND #4 | 165 |
|  | 6 | #5 AND TS=(randomi?ed OR placebo OR drug therapy OR randomly OR trial OR groups) | **RCTs: 140** |
| **Index to Taiwan Periodical Literature System** | 1 | (停經 + 更年期 + 絕經 + 卵巢早衰 + climacter* + menopaus* + preimenopaus* + perimenopaus* + postmenopaus* + anovulation* +潮紅 + 熱 + 汗 + flush* + ((hot + night* + nocturnal*) * (flash* + sweat*)) + vasomoto) * (針灸 + 電針 + 耳針 + 針藥 + 針刺 + 腹針 + 穴 + 電刺 + 經絡 + 灸 + 指壓 + 推拿 + acupunctur* + acupressure* + acupoint* + electroacupunctur* + meridian* + moxibustion* + auriculotherapy + TENS + PENS + stimulat* + electrostimulat*) | **13** |
